# *UNC5C*: Novel Gene Associated with Psychiatric Disorders Impacts Dysregulation of Axon Guidance Pathways

**DOI:** 10.3390/genes15030306

**Published:** 2024-02-27

**Authors:** Simone Treccarichi, Pinella Failla, Mirella Vinci, Antonino Musumeci, Angelo Gloria, Anna Vasta, Giuseppe Calabrese, Carla Papa, Concetta Federico, Salvatore Saccone, Francesco Calì

**Affiliations:** 1 Oasi Research Institute-IRCCS, 94018 Troina, Italy; streccarichi@oasi.en.it (S.T.); pfailla@oasi.en.it (P.F.); mvinci@oasi.en.it (M.V.); amusumeci@oasi.en.it (A.M.); agloria@oasi.en.it (A.G.); avasta@oasi.en.it (A.V.); pcalabrese@oasi.en.it (G.C.); cpapa@oasi.en.it (C.P.); cali@oasi.en.it (F.C.); 2 Department Biological, Geological and Environmental Sciences, University of Catania, Via Androne 81, 95124 Catania, Italy; concetta.federico@unict.it

**Keywords:** *UNC5C* gene, netrin 1/DCC, axon guidance, microtubule dynamics, whole-exome sequencing

## Abstract

The UNC-5 family of netrin receptor genes, predominantly expressed in brain tissues, plays a pivotal role in various neuronal processes. Mutations in genes involved in axon development contribute to a wide spectrum of human diseases, including developmental, neuropsychiatric, and neurodegenerative disorders. The NTN1/DCC signaling pathway, interacting with UNC5C, plays a crucial role in central nervous system axon guidance and has been associated with psychiatric disorders during adolescence in humans. Whole-exome sequencing analysis unveiled two compound heterozygous causative mutations within the *UNC5C* gene in a patient diagnosed with psychiatric disorders. In silico analysis demonstrated that neither of the observed variants affected the allosteric linkage between UNC5C and NTN1. In fact, these mutations are located within crucial cytoplasmic domains, specifically ZU5 and the region required for the netrin-mediated axon repulsion of neuronal growth cones. These domains play a critical role in forming the supramodular protein structure and directly interact with microtubules, thereby ensuring the functionality of the axon repulsion process. We emphasize that these mutations disrupt the aforementioned processes, thereby associating the *UNC5C* gene with psychiatric disorders for the first time and expanding the number of genes related to psychiatric disorders. Further research is required to validate the correlation of the *UNC5C* gene with psychiatric disorders, but we suggest including it in the genetic analysis of patients with psychiatric disorders.

## 1. Introduction

Psychiatric disorders are well defined in the *Diagnostic and Statistical Manual of Mental Disorders, Fifth Edition* (DSM-5). They can be classified into various diagnostic categories with continuous dimensional systems of classification, incorporating findings from neurobiological science into systems of diagnosis [1]. Remarkably, they are characterized by different natures encompassing various symptoms, such as mental impairments, depression, sleep disorders, as well borderline personality syndrome [2,3,4,5]. As documented in the PsyGeNET database, more than 2000 genes are associated with neuropsychiatric disorders [6]. In fact, it is widely recognized that psychiatric disorders can have a genetic correlation, with the inheritance of specific genetic variants capable of disrupting normal cognitive functions [7,8]. Even when individuals receive professional care, assessments are intermittent and may be limited partly due to the episodic nature of psychiatric symptoms [9].

As has been extensively documented, psychiatric disorders display a multifactorial origin. In fact, the intricate interplay between genetic and environmental factors during embryonic development may contribute to alterations in neurodevelopmental processes, potentially influencing the susceptibility to psychiatric disorders during the lifecycle [10,11,12,13]. Within this context, it is widely recognized that the onset of psychiatric disorders can manifest during childhood and adolescence, and they can be a prediction tool for future young-adult disorders [14].

In accordance with previous studies, mutations in genes operating in the axon guidance pathway are significantly linked to psychiatric disorders, encompassing schizophrenia, depression, obsessive compulsive disorder, and post-traumatic stress disorder [15,16,17].

Within this context, the axon guidance signaling mechanism involves Netrin 1 (NTN1) and its receptors Deleted in Colorectal Cancer (DCC) and Unc-5 Netrin Receptor C (UNC5). The NTN1/DCC complex has previously been linked to schizophrenia and neuropsychiatric disorders during adolescence [18,19,20].

Among the various gene families involved in neuronal development, the UNC-5 family of netrin receptors plays a pivotal role in various neuronal processes, including synaptic development and axon guidance. In fact, it is involved in presynaptic development, neuronal apoptosis, and migration in the cerebral cortex [21,22,23]. Within this context, it is crucial in spinal accessory motor neurons during the early stages of embryonic development [24].

As has been outlined by different studies, the *UNC5* gene family is predominantly expressed during the adolescence period, specifically within the puberty period [25,26,27]. In mice, the period of adolescence, characterized by high expression patterns of UNC5C, corresponds to 21 days of age [27]. In fact, adolescence represents a critical developmental phase characterized by profound changes in both behavior and brain anatomy. The prefrontal cortex, accountable for our most intricate cognitive functions, is still in the process of establishing connections during this period [28,29]. Mammals possess four UNC5 receptors in the cell membrane, UNC5A, UNC5B, UNC5C, and UNC5D, also known as UNC5H1, UNC5H2, UNC5H3, and UNC5H4, respectively. All of them are transmembrane receptors for netrin 1 (NTN1) in various brain tissues [30,31].

The UNC5 family of receptors comprises various domain patterns that facilitate significant structural changes in the protein. Specifically, the ZU5 domain plays a crucial role in achieving a supramodular protein structure [32]. These unique domain organizations, such as ZU5-UPA-DD in the cytoplasmic portion of UNC5, are also observed in ankyrins, an extensive family of scaffold proteins pivotal for assembling specialized membrane microdomains housing ion channels, cell adhesion molecules, and cytoskeletal components in various cells. Comparative analysis of the ZU5 domains across different proteins underscores the ZU5 domain’s compact and versatile nature as a protein interaction module [33,34].

Within this family, the receptors have the unique ability to initiate two contrasting intracellular signaling cascades based on the presence or absence of the ligand. As a result, these receptors are capable of orchestrating two opposing intracellular signals: one in the presence of the ligand and the other in its absence [35]. UNC5 signaling is involved in the pathogeneses of various cancers; in the absence of its ligand, Netrin-1, UNC5 proteins induce cell death, while, in the presence of the receptor, they actively promote cell survival [31,36,37]. Inhibition of the Netrin-1 ligand has been reported to decrease the invasiveness and angiogenesis of tumors [38,39]. Specifically, the downregulation or gene knockout of UNC5C significantly promotes tumor and metastasis growth both in vitro and in vivo [40,41,42]. The binding of NTN1/Netrin-1 has the potential to induce the dissociation of UNC5C from polymerized TUBB3 (Tubulin β 3 Class III) within microtubules through its cytoplasmic domain. This mechanism may result in heightened microtubule dynamics and subsequent axon repulsion, as previously demonstrated [43].

The UNC5B signaling pathway has also been reported to be involved in several processes, including neural development, developmental angiogenesis, and inflammatory processes [44]. *UNC5C* has been proposed as a candidate gene for Alzheimer’s disease (AD) in previous studies [45]. Additionally, several studies have reported that *UNC5C* is predominantly expressed in brain tissues [46,47,48,49].

**Figure 1:**
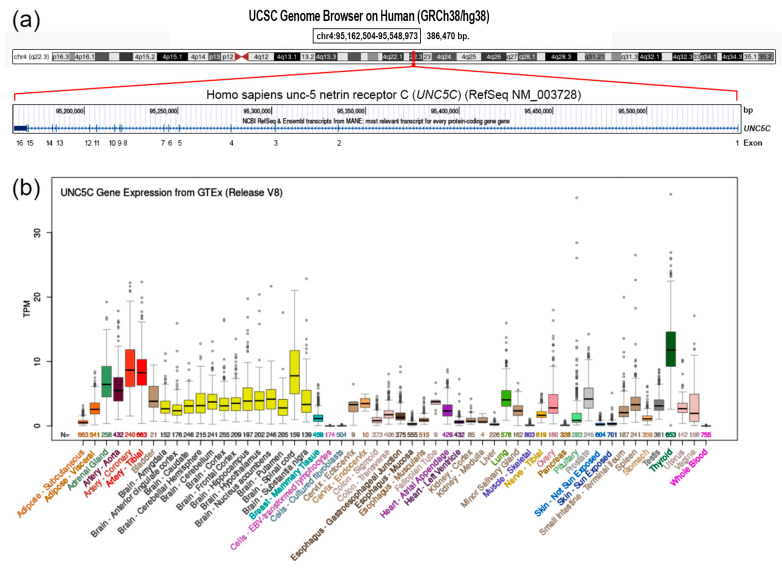
Genomic and transcriptional features of the *UNC5C* gene. (**a**) Genomic organization of *UNC5C* gene localized in chromosomal band 4q22.3 (upper part). Gene is transcribed from telomere to centromere side and consists of 16 exons (lower part). (**b**) Expression levels of *UNC5C* gene in healthy human tissues. Expression levels were obtained in 54 human tissues from GTEx RNA–seq of 17,382 samples from 948 donors (V8, August 2019). TPM: transcripts per million. Data and images from the UCSC Genome Browser (http://genome.ucsc.edu, accessed on 2 February 2024).

In the current study, we investigated a patient presenting with two compound heterozygous mutations in the *UNC5C* gene (Figure 1), hypothesizing that these mutations are causative of the diagnosed psychiatric disorders observed in the patient.

## 2. Materials and Methods

### 2.1. Library Preparation and NGS Analysis

Genomic DNA was extracted from peripheral blood leukocytes obtained from the patient and parents. DNA extraction was conducted in accordance with a previous protocol [50]. Whole-exome sequencing (WES) analysis was performed using Ion AmpliSeq™ Exome RDY Kits following the manufacturer’s instructions (Thermo Fisher Scientific, Waltham, MA, USA). The quality of the libraries was assessed using DNA 1000 chips on the Tape Station 4200 (Agilent, Santa Clara, CA, USA) and Qubit dsDNA BR Assay Kits (Invitrogen, Waltham, MA, USA). For the analysis, pooled libraries were employed for the emulsion PCR on the Ion Chef Instrument according to the manufacturer’s protocol (Thermo Fisher Scientific, Waltham, MA, USA). Finally, we sequenced each loaded Ion 550™ chip on the S5 System (Thermo Fisher Scientific, Waltham, MA, USA). A total of 98% of the regions of interest have a minimum coverage of at least 20×. The pathogenic variants were confirmed by conventional Sanger sequencing (Applied Biosystems Prism 3130 DNA Analyzer, Thermo Fisher Scientific, Waltham, MA, USA) using primers designed by the online tool available from the National Center for Biotechnology Information (NCBI) (https://www.ncbi.nlm.nih.gov/tools/primer-blast/, accessed on 10 October 2023).

### 2.2. Data Analysis

Data analysis was performed to assess the structure of the UNC5C protein using the UniProt database (https://www.uniprot.org/) (accessed on 14 November 2023) and UCSF ChimeraX (software developed by the Resource for Biocomputing, Visualization, and Informatics at the University of California, San Francisco, with support from National Institutes of Health R01-GM129325 and the Office of Cyber Infrastructure and Computational Biology, National Institute of Allergy and Infectious Diseases. https://www.cgl.ucsf.edu/chimerax/) for the high-quality protein visualization (accessed on 14 November 2023). All the common variants and non-exonic polymorphisms were excluded, keeping polymorphisms with a minor allele frequency (MAF) of <1% in the public databases: gnomAD Exomes v.3.1.2, the 1000 Genome Project, and the Exome Sequencing Project (accessed on 14 November 2023). The pathogenic variants were searched on the Human Gene Mutation Database (HGMD Professional 2023). VarAFT filtering (https://varaft.eu/, accessed on 30 October 2023) on vcf files was used. The variation found was classified according to the “American College of Medical Genetics” (ACMG) guidelines [51] and was performed with VarSome according to a previous study [52] and other evidence from the literature. In silico protein–protein interaction prediction was performed using PEPPI (https://zhanggroup.org/PEPPI/) and THREPP (https://zhanggroup.org/Threpp/) tools (both accessed on 10 December 2023). The in silico conservation rate was evaluated using the DOMINO website tool (www.ub.edu/softevol/domino, accessed on 10 December 2023), in accordance with previous research [53,54].

## 3. Results

### 3.1. Clinical Report

The patient was a 20-year-old male, the firstborn of non-consanguineous healthy parents with a negative family history of neurodevelopmental disorders. The mother reported no exposure to tobacco, drugs, or alcohol. The boy was born at term by emergency cesarean section due to gestosis, after a pregnancy complicated by threats of abortion during the first trimester and arterial hypertension. The birth weight was 2680 g. Furthermore, no signs of perinatal distress were reported. Independent walking was at 14 months, while the first words were observed at 10–12 months with subsequent normal language development. Sphincter check-up was at 3 years. At around the age of 4 years old, he began to complain of easy fatigue on long journeys and occasional muscle pain in his legs. At the age of 10 years old, he underwent a Neuropsychiatric Inventory (NPI) examination due to the presence of behavioral disorders characterized by easy irritability, poor tolerance to frustration, an oppositional–provocative pattern, and, at times, other-directed aggressive actions. A few days after this visit, he presented an episode of visual disperception (specifically, he saw black and red spots and shadows) and a “sensation of having people close to him”, which lasted about 3 h and resolved spontaneously.

In September 2018, the diagnosis of a panic attack was made. Several electroencephalograms (EEGs) in 2017–2021 resulted within normal limits. Brain magnetic resonance imaging (MRI), performed in November 2019, was found to be normal. The audiometric examination revealed “left normal hearing, medium-severe right sensorineural hearing loss at central and high frequencies”. An orthopedic examination revealed lumbar scoliosis (HP: 0004626) of the right-convex dorsal–lumbar spine with a marked costal hump on the bending test. No pathological movement patterns were evident. A reduction in the medial longitudinal arches of the feet was ascertained (in the past, he wore insoles for flat feet). The EEG, while the patient was awake, was free of paroxysmal anomalies. A new brain MRI, in 2021, was within normal limits. He showed normal cognitive ability as indicated by the Wechsler Adult Intelligence Scale IV (WAIS-IV) assessment, with specific scores as follows: intelligence quotient (IQ): 108; verbal comprehension index (VCI): 108; perceptual reasoning index (PRI): 96; processing speed index (PSI): 92; working memory index (WMI): 114. No learning problems were detected, but mathematical skills were insufficient.

During both interviews and performance assessments, an anxious response to difficulty was noted, accompanied by tendencies toward resignation, accompanied by a sense of discomfort, hand tremors, and sudden chaotic movements, all aimed at avoiding perceived failure, which was often only anticipated. A masked request for attention was observed during interviews through mildly provocative behaviors designed to solicit a specific response from the examiner. Furthermore, the mother reported peculiar episodes of severe tachycardia and a sensation of suffocation resembling panic attacks even during neutral moments and non-stressful situations. These issues were only occasionally observed and manifested in mild forms during the patient observations.

### 3.2. Next-Generation Sequencing

**Figure 2:**
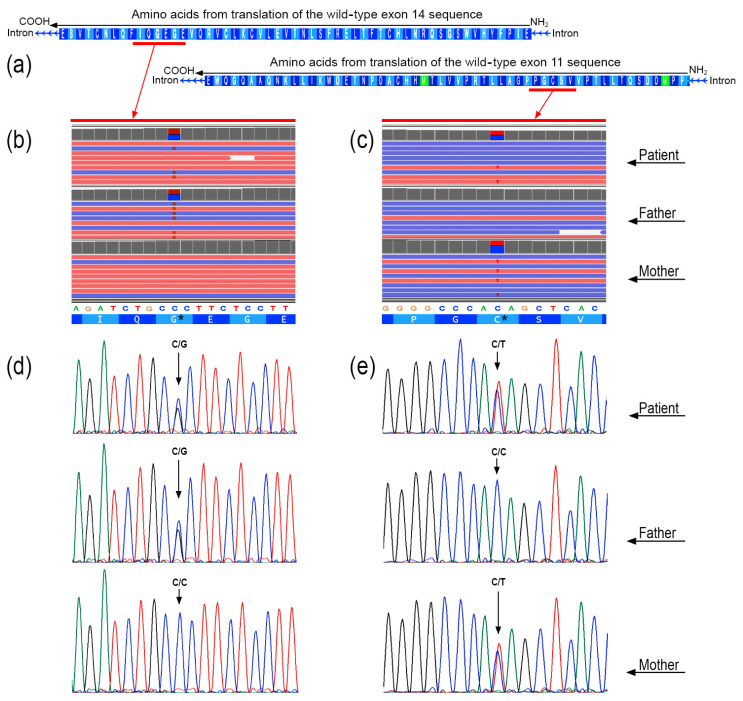
Next-generation sequencing results for observed variants in *UNC5C* gene. (**a**) Amino acid sequences corresponding to translation of DNA sequences from exon 11 and exon 14. Horizontal arrows above amino acidic sequences indicate NH_2_ → COOH direction. Modified from UCSC Genome Browser (http://genome.ucsc.edu, accessed on 2 February 2024). (**b**) Variant p.G806A and (**c**) variant p.C594Y detected by means of whole-exome sequencing (WES) of patient, father, and mother. Images were obtained by Integrative Genome Viewer (IGV). Asterisks, in the amino acid sequences, indicate mutated amino acids in *UNC5C* gene of subjects. (**d**,**e**) Variant confirmation through Sanger sequencing for both variants: p.G806A and p.C594Y. Two genotypic variants for each subject indicated in electropherograms. In the electropherograms, black, blue, green, and red profiles indicate G, C, A, and T nucleotide, respectively.

**Figure 3:**
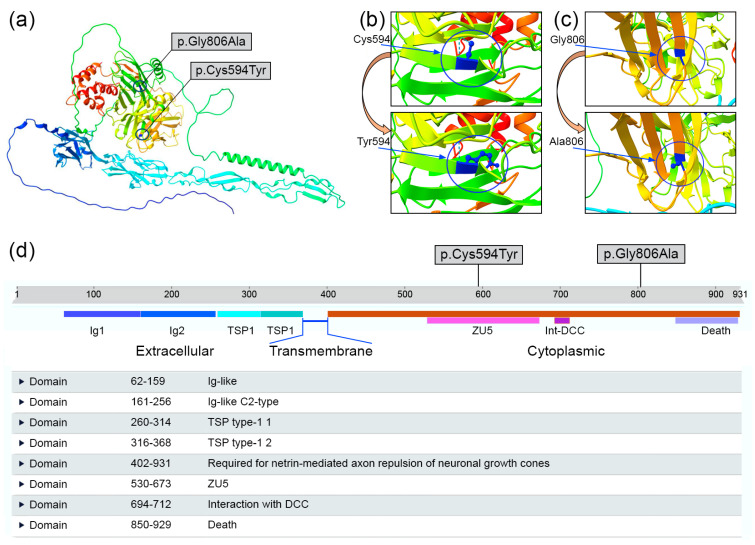
UNC5C wild-type protein and mutated forms. (**a**) Predicted protein structure of UNC5C using UCSF ChimeraX v.1.7 molecular modeling system. (**b**,**c**) Distinctions between wild-type (above) and mutated (below) amino acidic structures. Specifically, in (**b**), cysteine was replaced by hydrophobic amino acid tyrosine, resulting in a reduction of one hydrogen bond (from 2 to 1). Arrows indicate the position of the involved amino acids. (**d**) Functional domains within UNC5C protein, with affected domains by both identified mutations being ZU5 (530–673 aa) and region crucial for netrin-mediated axon repulsion of neuronal growth cones (402–931 aa).

Whole-exome sequencing (WES) did not identify potential causative variants in known genes associated with psychiatric disorders. However, it revealed the presence of two mutations within the *UNC5C* gene. Specifically, the first mutation identified in exon 11 at position c.1781C>T, resulting in the amino acid variation p.C594Y, was inherited from the mother. The second mutation, situated in exon 14 at position c.2417C>G, causing the protein variation p.G806A, was inherited from the father. Sanger sequencing confirmed the mutations, and specifically that p.C594Y was inherited from the mother, while G806A was inherited from the father (Figure 2). The patient exhibited the inheritance of both mutations, resulting in a compound heterozygous condition (Figure 3).

**Figure 4:**
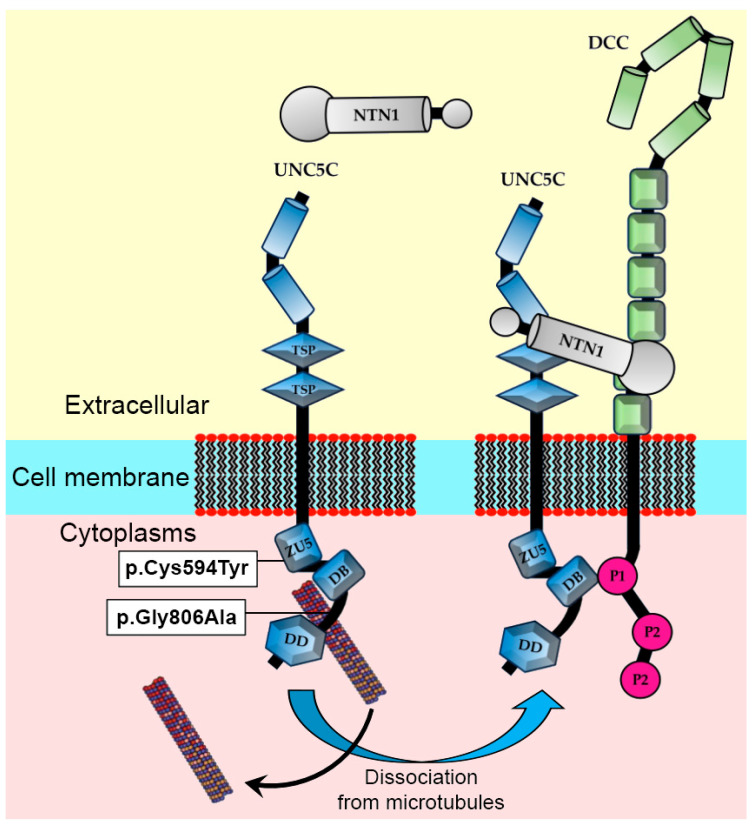
Graphical representation of UNC5C receptor localization, which is localized within phospholipidic membrane, illustrating allosteric linkage between UNC5C and complex netrin-1 (NTN1) with Deleted in Colorectal Cancer (DCC). Notably, both allelic variants were localized into cytoplasmic domains.

Precisely, the first mutation occurred in correspondence of the region required for the netrin-mediated axon repulsion of neuronal growth cones (RNRGCs) (from aa 402 to 931), also involving the ZU5 domain (from aa 530 to 673). Both allelic variants were observed within the supramodular structure pattern ZU5-DB (UPA)-DD (Figure 4). Conversely, the second mutation specifically affected only the domain involved in axon repulsion (from aa 402 to 931).

### 3.3. In Silico Prediction

In silico predictive tools unveiled a deleterious impact on the protein function using multiple prediction algorithms, as detailed in Table 1. Specifically, the variant p.Cys594Tyr was classified as “pathogenic moderate” by four tools (BayesDeladdAF, MetaRNN, FATHMM-XF, and MutPred). Conversely, it was classified as “pathogenic supporting” by five tools (EIGEN, EIGEN PC, FATHMM-MKL, LRT, and PrimateAI). Finally, it was classified as “uncertain” by nine tools (BayesDelnoAF, REVEL, LIST-S2, BLOSUM, CADD, DANN, Mutation assessor, MutationTaster, and MVP). The variant p.Gly806Ala was classified as “pathogenic moderate” by four predictive tools, precisely, BayesDeladdAF, MetaRNN, EIGEN, and EIGEN PC. Furthermore, it was classified as “pathogenic supporting” by five tools, specifically, MetaRNN, FATHMM-MKL, FATHMM-XF, LIST-S2, and LRT. Notably, eleven tools (BayesDelnoAF, REVEL, BLOSUM, CADD, DANN, Mutation assessor, MutationTaster, MutPred, MVP, PrimateAI, and SIFT4G) classified the variant as “uncertain”. Moreover, the PROVEAN algorithm classified both the observed variants as “deleterious”. Neither of these two variants were found in the Genome Aggregation Database (GnomAD v.3.1.2) among healthy individuals from the same ethnic group (European non-Finnish).

As was outlined by the DOMINO analysis, both the mutations showed the autosomal recessive inheritance pattern. STRING analysis and bibliographical references showed a robust correlation between UNC5C and the Netrin-1 (NTN1) gene. PEPPI and THREPP analyses indicated the absence of significant allosteric variations between both mutated UNC5C proteins and NTN-1, as compared to the wild-type protein (Table 2).

## 4. Discussion

### 4.1. UNC5C Gene Variant Identification

As has been extensively documented, psychiatric disorders show a multifactorial origin, encompassing both environmental and genetic factors. In fact, epigenetic alterations have a profound influence on gene translation and play a key role in brain development [11].

Whole-exome sequencing showed two mutations within the *UNC5C* gene. UNC5C acts as the receptor for netrin 1 and exhibits predominant expression in brain tissues, playing a crucial role in the axon guidance and repulsion processes. The molecular mechanisms involved in the axon guidance process are mediated by microtubules. Aberrations in genes operating in the axon guidance pathway have been significantly linked to psychiatric disorders [15,16,17]. Furthermore, the WES analysis did not reveal any potentially causative variants in genes associated with psychiatric disorders.

Specifically, the first amino acid variation, at 594 aa, was inherited from the father, while the second one, at 806 aa, was inherited from the mother. No psychiatric diseases were observed in either parent. The inheritance prediction analysis carried out by DOMINO showed an autosomal recessive (AR) inheritance pattern. It is well known that compound heterozygosity can significantly contribute to enhance the disease severity in psychiatric disorders [55,56,57,58,59]. These mutations are presumed to disrupt the interaction between the cytoplasmic functional ZU5 domain and the region required for the netrin-mediated axon repulsion of neuronal growth cones. In fact, in silico predictive analysis classified both the identified mutations as “probably damaging”, according to various algorithms (Table 1). Neither variant was found in the Genome Aggregation Database (GnomAD) among healthy individuals from the same ethnic group (European non-Finnish).

In the current manuscript, we are supposing that the two variants detected may contribute to the severity of the phenotype, in terms of psychiatric disorders. Notably, a Mendelian Inheritance in Man (MIM) number and code were not assigned for this gene. Based on these findings, we are establishing, for the first time, an association between the *UNC5C* gene and psychiatric disorders.

### 4.2. Impact of Observed Variants on Axon Guidance Signaling

As outlined in previous studies, UNC5C participates in axon guidance signaling, interacting with its ligand, NTN1, and another receptor named DCC. Within this context, it is worth mentioning that NTN1 has been identified as a candidate gene for psychiatric disorders, including depression [20,60,61]. Specifically, NTN1 is involved in the axon guidance process, enabling the adolescent expansion of mesocorticolimbic pathways, particularly the one related to dopamine release [25,62]. Remarkably, the use of in silico prediction tools confirmed that the two mutations detected have no impact on the protein–protein interaction between UNC5C and NTN1, as reported in Table 2. In fact, these mutations are located within crucial cytoplasmic domains, specifically ZU5 and the region required for the netrin-mediated axon repulsion of neuronal grown cones. We hypothesize that both variants impact the linkage between microtubules, operated by UNC5C cytoplasmic domains, leading to abnormalities in the axon guidance mechanism. The linkage with microtubules is mediated by the DCC receptor. In fact, the interaction among the UNC5C, NTN1, and DCC complex facilitates subsequent chemotropic transport along microtubules into the growth cone [43,63]. Consequently, we hypothesize that both allelic variants significantly alter this process, potentially contributing to the manifestation of severe psychiatric disorders in the patient. To further substantiate our hypothesis, a previous study in mice (*Mus musculus*) elucidated the intricate relationship between Netrin-1 and its receptor, UNC5C, during the dopamine axon guidance process toward the prefrontal cortex [27]. The study documented the disruption of a transient gradient, specifically occurring during adolescence, a critical developmental stage marked by significant changes in behavior and brain anatomy [27]. Furthermore, as previously outlined, UNC5C haploinsufficiency results in significant increases in tyrosine hydroxylase (TH) expression in the medial prefrontal cortex, with no parallel effect observed in the nucleus accumbens (NAc) [64]. As is well known, the NAc regulates multiple behaviors, and its dysfunction has been linked to many neural disorders, frequently observed during the adolescence growth stage [65]. In fact, the UNC5C protein plays a pivotal role in coordinating the assembly of neural circuits during adolescence development [64].

### 4.3. Impact of Observed Variants on Functional Cytoplasmic Domains

The UNC5 family of receptors possesses diverse domain patterns that facilitate significant structural changes in the protein. Notably, both allelic variants identified are situated in the cytoplasmic region, spanning the region crucial for the netrin-mediated repulsion of neuronal growth cones. In particular, the variant observed at position 594 within the UNC5C amino acid chain is located in the ZU5 domain. As previously documented, these domains, regulating the supramodular structure ZU5-UPA-DD, directly contribute to the axon repulsion process mediated by microtubules (Figure 4). These unique domain organizations, such as ZU5-UPA-DD in the cytoplasmic portion of UNC5, are also observed in ankyrins, an extensive family of scaffold proteins pivotal for assembling specialized membrane microdomains housing ion channels, cell adhesion molecules, and cytoskeletal components in various cells.

As previously mentioned, in silico analysis performed by various algorithms demonstrated that neither variant observed affected the allosteric linkage between UNC5C and NTN1 (Table 2). In fact, these mutations were located within crucial cytoplasmic domains, specifically ZU5 and the region required for the netrin-mediated repulsion of neuronal grown cones. These domains play a critical role in forming the supramodular protein structure and directly interact with microtubules, ensuring the functionality of the axon repulsion process [32,33,34,35].

In the current manuscript, we emphasize that both the observed variants can significantly impact the microtubule dynamics in the dysregulation of the axon guidance pathways. We propose, for the first time, an association between the *UNC5C* gene and psychiatric disorders, thereby expanding the understanding of genes related to psychiatric disorders.

### 4.4. Further Implications

UNC5C has been previously identified as a candidate gene for Alzheimer’s disease [45]. Consequently, we cannot exclude the possibility of a subsequent onset of Alzheimer’s disease in the examined patient, particularly during advanced age. In fact, the patient will be monitored over time to assess the severity of his psychological conditions.

Further functional analyses are necessary to validate the impact of these variants on the axon guidance mechanism. In this context, the utilization of in vitro studies and a larger cohort of patients are imperative to gain a more comprehensive understanding of the phenotype associated with both variants in UNC5C.

## 5. Conclusions

UNC5C acts as the receptor for netrin 1 and exhibits predominant expression in brain tissues, playing a crucial role in the axon guidance and repulsion processes during adolescence. Whole-exome sequencing uncovered two heterozygous variants, resulting in a compound heterozygosity condition in a patient exhibiting psychiatric disorders. These mutations are presumed to disrupt the interaction between the cytoplasmic functional ZU5 domain and the molecular mechanisms involved in the axon guidance process mediated by microtubules. In silico analysis suggests the likely pathogenic significance of these mutations, highlighting their autosomal inheritance. Further investigations are necessary to elucidate the correlation between UNC5C and proteins involved in the axon guidance and repulsion pathways.

## Figures and Tables

**Table 1:** In silico prediction of effects related to both variants identified within *UNC5C* gene (NM_003728).

	c.1781G>A (p.Cys594Tyr)		c.2417G>C (p.Gly806Ala)	
In silico Predictive tool	Prediction	Score	Prediction	Score
**BayesDeladdAF**	Pathogenic Moderate	0.2533	Pathogenic Moderate	0.2297
**MetaRNN**	Pathogenic Moderate	0.9091	Pathogenic Supporting	0.763
**MetaLR**	Benign Supporting	0.3355	Benign Supporting	0.3358
**MetaSVM**	Benign Supporting	−0.4082	Benign Supporting	−0.4582
**BayesDelnoAF**	Uncertain	0.1261	Uncertain	0.09217
**REVEL**	Uncertain	0.648	Uncertain	0.486
**EIGEN**	Pathogenic Supporting	0.8281	Pathogenic Moderate	0.8924
**EIGEN PC**	Pathogenic Supporting	0.7964	Pathogenic Moderate	0.8802
**M-CAP**	Benign Supporting	0.05996	Benign Moderate	0.01793
**SIFT**	Tolerated	0.095	Tolerated	0.109
**FATHMM-MKL**	Pathogenic Supporting	0.9848	Pathogenic Supporting	0.9882
**FATHMM-XF**	Pathogenic Moderate	0.9595	Pathogenic Supporting	0.927
**LIST-S2**	Uncertain	0.9707	Pathogenic Supporting	0.9807
**LRT**	Pathogenic Supporting	0	Pathogenic Supporting	0
**DEOGEN2**	Benign Supporting	0.05267	Benign Supporting	0.3835
**FATHMM**	Benign Supporting	0.77	Benign Supporting	0.7
**BLOSUM**	Uncertain	−6	Uncertain	−1
**CADD**	Uncertain	243.999	Uncertain	253.999
**DANN**	Uncertain	0.9969	Uncertain	0.9987
**Mutation assessor**	Uncertain	2.2	Uncertain	2.71
**MutationTaster**	Uncertain	1	Uncertain	1
**MutPred**	Pathogenic Moderate	0.813	Uncertain	0.537
**MVP**	Uncertain	0.8722	Uncertain	0.867
**PrimateAI**	Pathogenic Supporting	0.8076	Uncertain	0.7238
**PROVEAN**	Deleterious	−7.48	Deleterious	−4.16
**SIFT4G**	Benign Supporting	0.115	Uncertain	0.046
**Polyphen2**	Probably damaging	0.993	Probably damaging	0.999
**GnomADEuropean** ***Allele Frequency***	Not found		Not found	

**Table 2:** Prediction scores of protein–protein interaction between UNC5C and Netrin-1 (NTN-1).

Protein	LogLR (a)	Threpp Score (b)
UNC5C wild type	1.365	13.803
UNC5C p.C594Y	1.303	13.803
UNC5C p.G806A	1.299	13.803

(a) Logarithmic variation in likelihood ratio (logLR); (b) Threpp score from protein–protein interaction between UNC5C and Netrin-1 (NTN-1). Prediction was conducted by PEPPI (https://zhanggroup.org/PEPPI/) and THREPP (https://seq2fun.dcmb.med.umich.edu/Threpp/) tools, accessed on 10 December 2023, considering wild-type protein in addition to both detected mutations.

## Data Availability

The data presented in this study are available upon request from the corresponding author. The data are not publicly available to privacy of the patient and the family.
